# Lipid Biomarker and Carbon Stable Isotope Survey on the Dallol Hydrothermal System in Ethiopia

**DOI:** 10.1089/ast.2018.1963

**Published:** 2019-12-04

**Authors:** Daniel Carrizo, Laura Sánchez-García, Nuria Rodriguez, Felipe Gómez

**Affiliations:** Centro de Astrobiología (CSIC-INTA), Torrejón de Ardoz, Madrid, Spain.

**Keywords:** Lipid biomarkers, Bulk stable isotopes, Polyextreme environments, Limits of life, Dallol hydrothermal system

## Abstract

The remote Dallol Hot Springs, an active hydrothermal system in the volcanic region of Danakil (Ethiopia), is an interesting yet poorly studied polyextreme environment for investigating the limits of life. Here, we explored the presence of signs of life in five samples of sinter deposits at Dallol, by means of lipid biomarkers and stable isotope composition. The results reveal the existence of biological material with predominance of (presently or recently active) microbial sources, according to the relative abundance of low-over-high molecular weight moieties (*n-*alkanes, *n-*carboxylic acids, or *n-*alkanols), and the detection of diverse microbial-diagnostic compounds (*i.e.,* monomethyl alkanes; C_16:1_ ω7, C_18:1_ ω9, C_18:1_ ω10, C_18:2_ ω6,9 and *iso/anteiso* C_15_ and C_17_ carboxylic acids; or short-chained dicarboxylic acids). The molecular lipid patterns at Dallol suggest a microbial community largely composed of thermophilic members of the Aquificae, Thermotogae, Chroroflexi, or Proteobacteria phyla, as well as microbial consortia of phototrophs (*e.g.,* Cyanobacteria-Chloroflexi) in lower-temperature and higher-pH niches. Autotrophic sources most likely using the Calvin cycle, together with the acetyl coenzyme A (CoA) pathway, were inferred from the depleted bulk δ^13^C ratios (−25.9/−22.6‰), while sulfate-reducing bacteria were considered according to enriched sulfate (7.3/11.7‰) and total sulfur (20.5/8.2‰) δ^34^S ratios. The abundance of functionalized hydrocarbons (*i.e., n*-carboxylic acids and *n*-alkanols) and the distinct even-over-odd predominance/preference on the typically odd *n*-alkanes (CPI_alkanes_ ≤ 1) pointed to active or recent microbial metabolisms. This study documents the detection of biosignatures in the polyextreme environment of Dallol and raises the possibility of finding life or its remnants in other remote locations on Earth, where the harsh environmental conditions would lead to expect otherwise. These findings are relevant for understanding the limits of life and have implications for searching for hypothetical life vestiges in extreme environments beyond Earth.

## 1. Introduction

Hydrothermal systems have great significance in the early evolution of the biosphere. They are thriving ecosystems containing thermophilic microorganisms similar to those that existed early in Earth's history (Ward *et al.,* 1989). While deep-sea hydrothermal vents have been traditionally postulated to be the environment where life started out, recent geological, chemical, and computational findings rather point to a land-based alternative scenario (Damer, 2016, and references therein). According to this theory, a system of volcanic pools and hot springs on land provides, apart from the basic ingredients for life (*i.e.,* energy and nutrients), a way to create complex molecules and bring them together to promote prebiotic reactions (Van Kranendonk *et al.,* 2017). The alternation of drying and wetting spells combined with the continuous heat supply results in the formation of complex molecules (*i.e.,* polymers) from simpler units such as amino acids or fatty acids (Deamer and Georgiou, 2015). These systems have been operating on Earth for very long periods, as documented in the oldest known subaerial hydrothermal deposits in Australia (Djokic *et al.,* 2017). This land-based perspective is relevant from an astrobiological point of view because it guides scientists to different places in the Solar System to search for life beyond Earth. The examination of geothermal areas and their microbial community may contribute to decipher the origin and expansion of life on Earth and beyond, for instance in analogous hydrothermal systems on Mars (*e.g.,* Gusev Crater) or on Jupiter's and Saturn's icy moons (*e.g.,* Europa and Enceladus, respectively).

Subaerial thermal springs are important ecosystems not only as hosts of life but as long-term preservers of biosignatures (Djokic *et al.,* 2017). Microorganisms representative of early-evolved lineages of chemosynthetic life inhabit modern hot springs (Ward *et al.,* 1989), occurring as planktonic cells in fluids and as biofilms on the surface on interior fractures of mineral deposits (Pancost *et al.,* 2005). These microbial communities are largely composed of thermophiles, majorly inhabiting vent areas or occupying lower-temperature niches, such as hot-spring discharge channels and aprons (Campbell *et al.,* 2015a). The mineral entombment of biofilms and microbial mats living on the hydrothermal deposits facilitates the preservation of numerous microbial biosignatures (Cady *et al.,* 2003; Ruff and Farmer, 2016). In ancient thermal springs, the preservation of biological signatures provides great paleobiological and paleoenvironmental information for understanding early life (Knoll and Walter, 1996).

The search for molecular evidence of life is crucial for understanding the emergence and evolution of life on Earth and other Solar System bodies. Learning about habitability in other planetary bodies requires a deep knowledge of adaptability and life boundaries on Earth. Remote and inhospitable environments on Earth provide excellent settings for assessing the capability of the most resistant forms of life (extremophiles) to endure and thrive in the harshest conditions. The presence of life or its remnants has been investigated in diverse extreme environments on Earth (geothermal regions, hypersaline desert, acidic rivers, hyperarid frozen soils, deep caves, etc.), where life adapts to thrive in a variety of hostile conditions such as hypersalinity (*e.g.,* Cheng *et al.,* 2017; Sánchez-García *et al.,* 2018), aridity (*e.g.,* Wilhelm *et al.,* 2017), acidity (Fernández-Remolar *et al.,* 2005; Fernández-Remolar and Knoll, 2008), thermal systems (Farmer and Des Marais, 1999; Cady *et al.,* 2003; Sánchez-García *et al.,* 2019), or subzero temperatures (Rivkina *et al.,* 2007; Steven *et al.,* 2008). However, although it is well known that life can tolerate or even thrive under extreme conditions (Rothschild and Mancinelli, 2001), the impact of multiple physicochemical factors on the development of life is poorly understood (Harrison *et al.,* 2013). What are the limits of life and to what extent is life able to thrive in environments holding several extreme conditions at the same time are issues that need to be investigated.

**FIG. 1.**
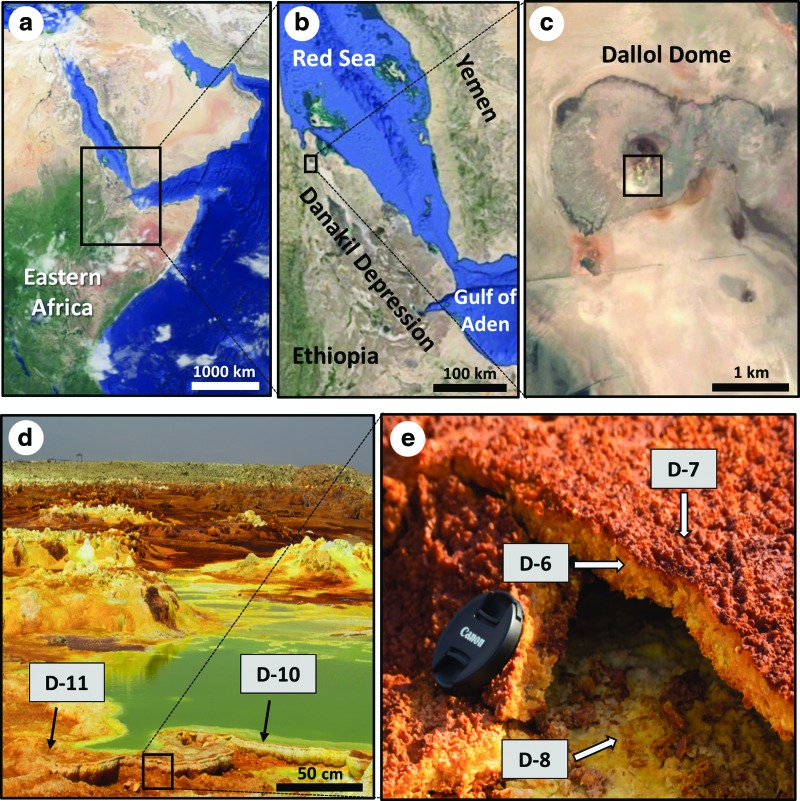
Map of eastern Africa (**a**), showing the Danakil Depression in Ethiopia (**b**), where the Dallol Hot Springs are located (**c**). An overview of the survey area with the three sampling sites (an active fumarole chimney, a terrace of evaporitic precipitates, and a hydrothermal pool) is shown (**d**). A close-up of the three evaporite samples from the small fumarole chimney is also included (**e**). The maps are shown as satellite images from Google Maps.

Biological studies on polyextreme environments are scarce (Ngugi *et al.,* 2016; Pérez *et al.,* 2018; Wierzchos *et al.,* 2018), with only a few sites, such as the Chilean Altiplano or Red Sea brines, investigated from that perspective. In the Danakil Depression, in northeast Ethiopia (Fig. 1), the volcanic features of the Erta Ale range at the Afar Triangle have created a polyextreme hydrothermal system. Categorized as hot desert climate, Danakil is considered one of the driest (annual precipitation between 50 and 100 mm; Garland, 1980) and hottest (mean annual temperature of 35°C; Fazzini and Bisci, 2015) places on the planet. Located in one of the most remote, inhospitable, and poorly studied regions in the world (*i.e.,* Danakil), Dallol is a complex and active hydrothermal system (Fig. 1c) composed of diverse hot springs that open into an arid desert. In Dallol, seawater and hydrothermal fluids mix, resulting in a hypersaline environment, where the springs discharge extremely hot (temperature from 90°C to 108°C; Franzson *et al.,* 2015; Kotopoulou *et al.,* 2019), oxygen-free, hyperacidic (pH ranging from −1.7 to 4; Gebresilassie *et al.,* 2011; Kotopoulou *et al.,* 2019), Fe-rich hydrothermal brines, which are halite supersaturated as soon as they are in contact with the atmosphere (Kotopoulou *et al.,* 2019). The heat and aridity in Dallol give rise to the development of large evaporitic deposits of about 1000 m depth that are rich in K, Mn, Fe, Mg, or Zn (Tadesse *et al.,* 2003). The abundance of metals makes Dallol Hot Springs an important area for mining exploitation (*e.g.,* rock salt, potassium salts, or manganese deposits) and trading (*e.g.,* Gebresilassie *et al.,* 2011; Darrah *et al.,* 2013; Franzson *et al.,* 2015). In addition, geotourism based on geothermal spring and volcano visiting is becoming more popular in the area (Erfurt-Cooper and Cooper, 2010), where the Dallol Springs are some of the major attractions because of their stunning colored waters, mineral salts, and landforms (Edelman and Roscoe, 2010). The scarce studies existing on Dallol are mostly focused on geological (Nobile *et al.,* 2012; Darrah *et al.,* 2013) and geophysical (Hovland *et al.,* 2006; Carniel *et al.,* 2010) interests related to the recent seismicity and volcanic activity, with a few works reporting on the hydrochemistry operating in this geothermal system (Gonfiantini *et al.,* 1973; Kotopoulou *et al.,* 2019). Little is known about the ecology and biochemistry in the polyextreme environment, with the only biological studies focused on studying the diversity or genome sequencing of halophilic microorganisms on industrially processed, and thus likely human and environmentally contaminated, samples (commercial salts) from Dallol (Gibtan *et al.,* 2016, 2017). There is no study that we know exploring the autochthonous distribution of microbial populations at Dallol. If microorganisms are present in the polyextreme hydrothermal brines and evaporitic fields, their existence would expand the limits of life supporting habitability on Earth and analogous extraterrestrial sites, thus rendering Dallol a site of unique astrobiological significance.

This work aimed to investigate the presence of biomarkers in evaporitic deposits of the Dallol Hot Springs to assess the habitability and/or preservation of biomolecules in the polyextreme environment. A geochemical approach based on the combination of lipid and bulk isotopic (δ^13^C and δ^32^S) analysis was proposed to assess the presence of life or its remnants in the inhospitable Dallol region. The distribution of lipid molecules with source-diagnosis value is analyzed to infer biological sources and assess activity level. The presence of lipids and isotopic biosignatures is interpreted in a mineralogical context. This study is framed within the UE-funded Europlanet Project (H2020), which, among other activities, investigates extreme environments for detecting biosignatures in very harsh conditions for life to exist, and seeks to validate those extreme environments as terrestrial analogs of other planets (*e.g.,* Mars, Jupiter's icy moon Europa).

### 1.1. Field settings

The present study is located in the Danakil Depression (Fig. 1), one of the hottest and most tectonically active regions in the world (Darrah *et al.,* 2013). The Danakil Depression is located near the triple junction of the Red Sea, Gulf of Aden, and the Ethiopian Rift, and is part of the East African Rift System, an active continental drifting between Africa and Arabia (Ebinger *et al.,* 2008), which causes the floor of the Danakil Depression to be located about 120 m below sea level (Holwerda and Hutchinson, 1968). From a geological point of view, the Danakil Depression is composed of three formations alternating as follows, from west to east: (a) Neoproterozoic metavolcanic and metasedimentary rocks, (b) Quaternary alluvial deposits intercalated with red beds, and (c) evaporites mainly consisting of halite and potash deposits, as well as sulfur (Gebresilassie *et al.,* 2011). The Neoproterozoic setting includes the mount Dallol and the surrounding hot-spring area (Fig. 1c), where the present study was conducted. The area around Dallol is occupied by an ephemeral salt lake sitting atop a 2 km thick evaporite sequence (Behle *et al.,* 1975) formed when an arm of the Red Sea was isolated by an uplifted horst block, causing almost complete evaporation of the water (Beyene and Abdelsalam, 2005). The evaporite sequence is dominated by halite and sylvite, and subordinate layers rich in carnallite and kainite (Holwerda and Hutchinson, 1968).

The volcanic heat causes the ascent of hot water through the layers of halite and anhydrite that dissolve and deposit over the basaltic lava flows, originating fumaroles, subaerial and subaqueous hydrothermal springs, and acidic brines that produce salt chimneys, pillars, hornitos, terraces, and pools of ephemeral colors (Carniel *et al.,* 2010; Kotopoulou *et al.,* 2019). The broad color palette of the landscape is one of the most striking features of Dallol, as colors range from pale green to dark brown and reds, due to the combined action of the continuous discharge of oxygen-free Fe(II)-rich spring brines, the low solubility of oxygen in high temperature, hyperacidic brines, and hypersaline brines, and therefore the slow oxidation of the Fe(II) species (Kotopoulou *et al.,* 2019). Three principal evaporitic formations in the Dallol hydrothermal system are (a) pillars, or circular columns composed of salt formed from outflows of water supersaturated with sodium chloride; (b) circular manifestations formed by deposition episodes, where mineral-supersaturated water begins precipitating out upon cooling, exsoluting and disintegrating material (*i.e.,* halides becoming richer in anhydrite and sulfur over time); and (c) acid pools (pH ∼0.7 to 4) derived from the oxidation of geothermal hydrogen sulfide to sulfuric acid upon mixing with groundwater (Franzson *et al.,* 2015). Our study focused on the acidic pools of the main Dallol hydrothermal area (14°14′19″N, 40°17′38″E) and their associated evaporites (Fig. 1c, 1d). The active subsidence in Dallol causes the delivery of salts coming from the subsurface hot water, which precipitate and form evaporitic structures, such as small chimneys surrounded by pools of hot water (Fig. 1d), enriched in metals and colorful precipitates of halite (NaCl), pyrolusite (MnO_2_), chlorargyrite (AgCl), wurtzite (ZnS), and iron-rich salts (Master, 2013).

## 2. Materials and Methods

### 2.1. Sample collection

In January 2016, geological samples were collected from three evaporitic sites in the Dallol Hot Springs (*ca.* 146 m below sea level), as part of a Europlanet sampling campaign (Grant agreement N° 654208). Three sampling locations were chosen aiming to cover different hydrothermal environments: an active fumarole chimney, an inactive terrace of evaporitic precipitates, and a hydrothermal pool. Five samples were collected in total (Fig. 1d, 1e). Salt precipitates were sampled from three different parts of the fumarole chimney: yellow (D6) and brownish (D7) precipitates from the outer and inner part of the chimney top layer, and yellow precipitates from the base of the fumarole structure (D8). One sample was taken from the yellow precipitates lying in the terrace nearby the fumarole chimney (D11), and another was taken of yellow-greenish precipitate from the edge of the green thermal pool (D10). Temperature and pH in the water pool were measured at 90°C and 2, respectively. The pH of the sampled material was measured to be around 4 in the five sites, whereas the *in situ* temperature was not measured but assumed to be in between atmospheric (diurnal range from 25°C to 45°C; Garland, 1980) and that measured in the water (90°C). The five evaporite samples were collected with a solvent-clean (dichloromethane and methanol) stainless-steel spatula, stored in polypropylene containers at −20°C, and, once in the laboratory, freeze-dried and ground in a pestle prior to lipid analysis.

### 2.2. Geochemical and mineralogical analyses

The mineralogical composition of the evaporites was measured with a Bruker X-ray diffractometer (AXS D8-Focus, XRD). The freeze-dried samples were ground and scanned in the 2·θ-diffraction angle from 5° to 70°, with a scanning step size of 0.01°, at 40 kV and 40 mA with a Cu X-ray source (Cu Kα1,2, λ = 1.54056 Å).

Anions and low-molecular-weight organic acids were measured by ion chromatography (IC) in the water-extractable phase of the samples. For this analysis, 2 g of sample was sonicated (3 × 1 min cycles) and diluted in 10 mL of deionized water, then filtered through a 22 μm GF/F. The filtrates were collected and loaded into a Metrohm 861 Advanced compact ion chromatograph (Metrohm AG, Herisau, Switzerland) undiluted or at dilution values, depending on ion concentrations. For all the anions, the column Metrosep A supp 7−250 was used with 3.6 mM sodium carbonate (NaCO_3_) as eluent. The pH of the water solutions was measured with a pH meter (WTW, GmbH & Co. KG, Weilheim, Germany) after 24 h of solution stabilization.

### 2.3. Lipid extraction, fractionation, and analysis

The evaporitic samples were Soxhlet extracted (∼30 g) with a mixture of dichloromethane/methanol (DCM:MeOH, 3:1, v/v) for 24 h, after addition of three internal standards (tetracosane-D_50_, myristic acid-D_27_, 2-hexadecanol). The total lipid extract (TLE) was concentrated to *ca.* 2 mL by using rotary evaporation, and activated copper was added to remove elemental sulfur. TLE was separated into polarity fractions by using Bond-Elut (bond phase NH_2_, 500 mg, 40 μm particle size) and Al_2_O_3_ (activated, neutral, 0.05–0.15 mm particle size) columns (Supplementary Fig. S1, https://www.liebertpub.com/suppl/doi/10.1089/ast.2018.1963). First, the neutral and acidic lipid fractions were obtained by eluting the TLE through a Bond-Elut column with 15 mL of DCM:2-propanol (2:1, v/v) and 15 mL of acetic acid (2%) in diethyl ether, respectively. Then, a further separation of the neutral lipid fraction into nonpolar and polar subfractions was done using 0.5 g of Al_2_O_3_ in a Pasteur pipette (*ca.* 2.5 cm high). The nonpolar fraction was obtained by eluting 4.5 mL of hexane/DCM (9:1, v/v), and the polar fraction by subsequently eluting 3 mL of DCM/methanol (1:1, v/v). The acidic and polar fractions were trans-esterified (BF_3_ in methanol) and tri-methylsilylated (N,O-bis(trimethylsilyl)trifluoroacetamide, BSTFA), respectively, and then analyzed as fatty acid methyl esters (*i.e.,* FAMEs) and trimethylsilyl (*i.e.,* TMS) alkanols by gas chromatography–mass spectrometry (GC-MS).

The three lipid fractions (*i.e.,* nonpolar, polar, and acidic) were analyzed by GC-MS using a 6850 GC system coupled to a 5975 VL MSD with a triple axis detector (Agilent Technologies) operating with electron ionization at 70 eV and scanning from *m/z* 50 to 650. The analytes were injected (1 μL) and separated on a HP-5MS column (30 m × 0.25 mm i.d. × 0.25 μm film thickness) with He as a carrier gas at 1.1 mL min^−1^. For the nonpolar fraction, the oven temperature was programmed from 50°C to 130°C at 20°C min^−1^, then to 300°C at 6°C min^−1^ (held 20 min). For the acidic fraction, the oven temperature was programmed from 70°C to 130°C at 20°C min^−1^ and to 300°C at 10°C min^−1^ (held 10 min). For the polar fraction, the oven temperature program was the same as for the acidic fraction, but the oven was held for 15 min at 300°C. The injector temperature was 290°C, the transfer line 300°C, and the MS source 240°C. Compound identification was based on the comparison of mass spectra and/or reference materials and quantification on the use of external calibration curves of *n*-alkanes (C_10_ to C_40_), *n*-FAMEs (*i.e.,* C_8_ to C_24_), *n*-alkanols (C_10_, C_14_, C_18_, and C_20_), and branched isoprenoids (2,6,10-trimethyl-docosane, crocetane, pristane, phytane, squalane, and squalene), all from Sigma-Aldrich. The recoveries of the internal standards averaged 72 ± 23 %.

### 2.4. Stable isotopic analysis of organic carbon, sulfate, and total sulfur

The stable isotopic composition of the bulk organic carbon, sulfate, and total sulfur was determined by isotope ratio mass spectrometry (IRMS), with a MAT 253 (Thermo Fisher Scientific). The isotopic analyses were conducted according to the respective USGS methods for carbon (Révész *et al.,* 2012a), sulfur in sulfate, (Kester *et al.,* 2011), and total sulfur (Révész *et al.,* 2012b). For organic carbon and total sulfur, 2 g of sample was ground and homogenized with a corundum mortar and pestle. The samples were decarbonated with concentrated HCl (37%) and, given the abundance of NaCl reported by IC (>98%), washed with deionized water to eliminate the dissolved salts through GF/F filtering (0.7 μm pore size, Whatman). Precombusted filters (8 h at 450°C) were dried in an oven (50°C) and analyzed by IRMS. For sulfate analysis, approximately 1 g of the sample was extracted with deionized water (20:1), by shaking for several hours. The supernatant was then extracted again with a solution of preheated HCl (1 M, 70°C) to ensure a complete extraction of the sulfate from the sample. The sulfate concentration is measured by IC. The pH of the extracted solution was adjusted to less than 2, and sulfate was then precipitated by using a saturated solution of barium chloride (BaCl_2_). The solution was allowed to stand overnight and then filtrated to collect the formed BaSO_4_. After drying in an oven at 50°C, the BaSO_4_ was analyzed for the isotopic composition of the sulfate by using the method by Kester and coauthors (2011).

The carbon and sulfates/sulfur results were reported as δ^13^C (abbreviation from δ[^13^C/^12^C]) and δ^34^S (abbreviation from δ[^34^S/^32^S]) values relative to the Pee Dee Belemnite limestone standard (PDB) and Vienna-Canyon Diablo Troilite (VCDT), respectively, in the usual parts per mill (‰) notation. An analytical precision of 0.1‰ was determined by using three certified standards for carbon (USGS41, IAEA-600, and USGS40), three for sulfur on sulfate (IAEA-SO5, IAEA-SO6, and NBS 127), and three for sulfur (IAEA-S1, IAEA S-2, and IAEA-S3). The total organic carbon content (TOC %) was measured with an elemental analyzer (Flash HT, Thermo Fisher Scientific), during the stable isotope measurements.

## 3. Results

### 3.1. Mineralogy and bulk geochemistry

**FIG. 2.**
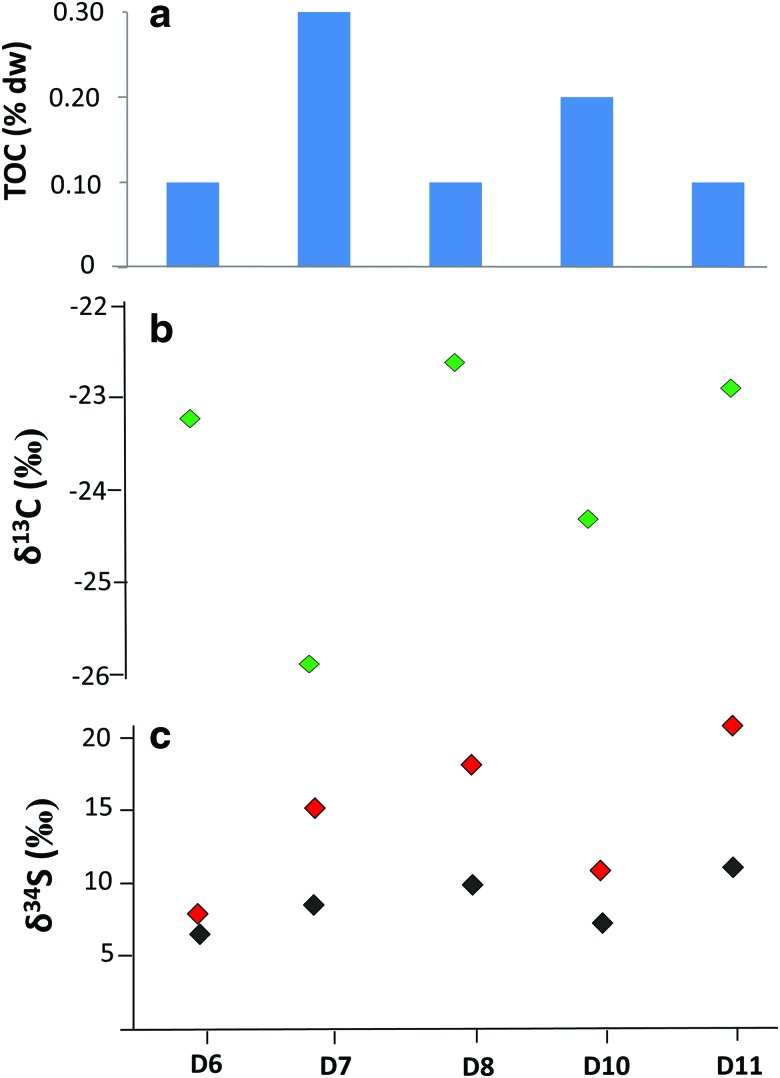
Geochemical composition of the bulk organic fraction in the Dallol samples; (**a**) concentration of total organic carbon (% of the sample dry weight), (**b**) stable carbon isotopic composition of organic carbon (‰ PDB), and (**c**) stable sulfur isotopic composition of total sulfur (red) and sulfur from sulfate (black) (‰ VCDT).

The XRD analysis indicated that the sample mineralogy majorly consisted of halite (NaCl, >90%), with lower presence of hydrothermal minerals such as bromian chlorargyrite (Ag(Cl,Br)) and wurtzite (ZnS). The only anions measured by IC were chloride (2215 ± 115 mg/g), fluoride (54 ± 3.4 μg/g), and sulfate (14 ± 0.83 mg/g), whereas no organic anions were detected. The TOC content was measured to vary from 0.12% to 0.35% of dry weight (dw) (Fig. 2a). The sample isotopic composition ranged from −22.6‰ to −25.9‰ for δ^13^C (Fig. 2b), from 6.5‰ to 11.7‰ for δ^34^S from sulfate (Fig. 2c), and from 8.2‰ to 20.5‰ for δ^34^S for total sulfur (Fig. 2c).

### 3.2. Lipid biomarkers

**FIG. 3.**
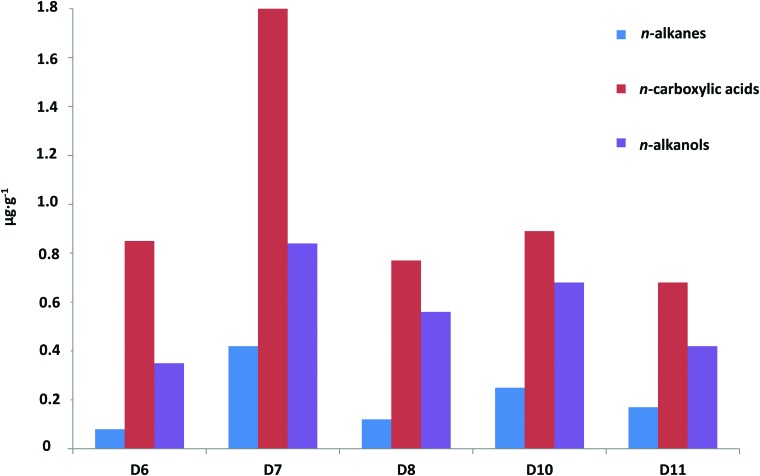
Relative concentration (μg·g^−1^) of the three major lipid families in the Dallol evaporites, the straight chain *n*-alkanes, *n*-carboxylic acids, and *n*-alkanols.

**FIG. 4.**
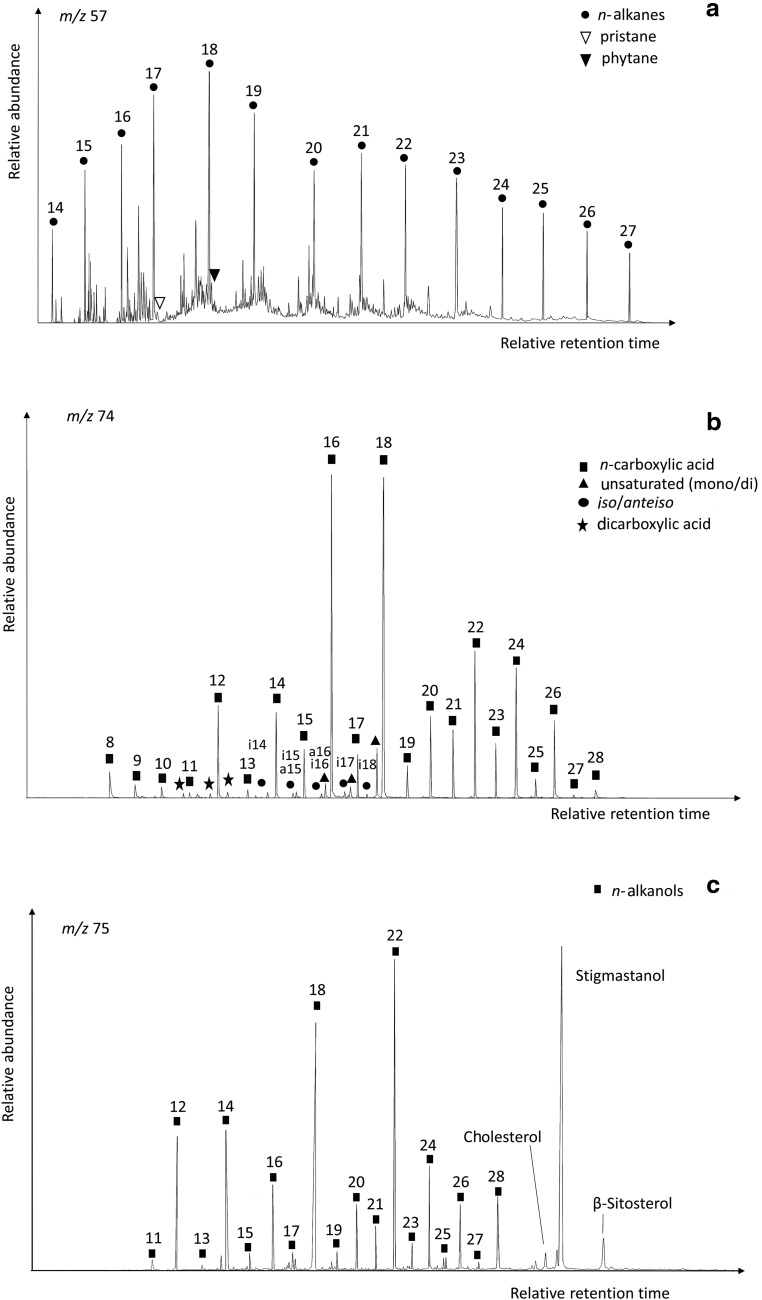
Mass chromatograms of the three major lipid families in the Dallol sample D7; *n*-alkanes (*m/z* 57) (**a**), carboxylic acids as methyl esters (*m/z* 74) (**b**), and *n*-alkanols as trimethyl-silyl esters (*m/z* 75) (**c**).

The GC-MS analysis of the lipid extracts detected the presence of diverse families (Table 1) using mass-to-charge ratios (*m/z*) of 57 (*n*-alkanes and isoprenoids), *m/z* = 74 (*n*-carboxylic acids, including saturated, unsaturated, and branched moieties), and *m/z* = 75 (*n*-alkanols and sterols) (Tables 2–4). Among all families, the *n*-carboxylic acids were the most abundant lipid compounds, followed by the *n*-alkanols, and *n*-alkanes (Fig. 3). The three major lipid families showed a clear even-over-odd predominance/preference. Straight-chain alkanes (aka *normal*, or *n*-alkanes) ranging from 14 to 27 carbons (C_14_ to C_27_) were measured at concentrations from 0.08 to 0.42 μg·g^−1^ (Tables 1 and 2). The *n*-alkane molecular distribution exhibited an average chain length (ACL) of 19–20 (Table 1) and a bimodal pattern with a maximum peak at C_18_ and a secondary peak at C_21_ (Fig. 4a). The relative abundance of low-molecular-weight (LMW) over high-molecular-weight (HMW) *n*-alkanes resulted in LMW/HMW ratios always larger than one (1.6–3.1; Table 1). Branched alkanes (mono-, di-, tri-, and tetramethyl congeners) were also found in the *m/z* = 57 ratio, with the monomethyl alkanes being the most abundant (Table 2). Among the branched alkanes, pristane (Pr) and phytane (Ph) were found (Fig. 4a) at concentrations from 0.05 to 0.10 μg·g^−1^ and from 0.02 to 0.07 μg·g^−1^, respectively (Table 2). Other isoprenoids such as squalane or crocetane were not detected.

**Table 1.** Concentration (μg·g^−1^) and Compositional Distribution of Lipid Biomarkers and Bulk Stable Isotopes in the Dallol Samples

	*D6*	*D7*	*D8*	*D10*	*D11*
TOC (% dw)	0.12	0.35	0.15	0.22	0.14
δ^13^C_OC_ (‰)	−23.2	−25.9	−22.6	−24.3	−22.9
δ^34^S total (‰)	8.2	15.3	18.4	10.9	20.5
δ^34^S SO_4_ (‰)	6.5	8.5	9.5	7.3	11.7
*n*-alkanes	0.08	0.42	0.12	0.25	0.17
branched *n*-alkanes^a^	0.05	0.25	0.09	0.16	0.12
ACL *n*-alkanes^b^	19	20	19	20	20
LMW*n*-alkanes/HMW*n*-alkanes^c^	3.10	1.62	2.46	1.82	1.53
CPI *n*-alkanes (c_14_–C_27_)^d^	0.86	1.05	0.98	0.98	1.01
pristane^e^	0.05	0.10	0.06	0.07	n.d.
phytane^f^	n.d.	0.07	0.03	0.02	n.d.
*n*-carboxylic acids	0.85	1.77	0.78	0.88	0.68
unsaturated carboxylic acids^g^	0.12	0.15	0.12	0.13	0.10
dicarboxylic acids	0.02	0.02	0.01	0.01	0.01
*iso*/*anteiso*-carboxylic acids^h^	0.01	0.05	0.02	0.02	0.01
MM-carboxylic acids^i^	n.d.	0.02	0.01	0.03	0.01
Σ carboxylic acids^j^	1.01	2.01	0.93	1.07	0.82
ACL *n*-carboxylic acids^b^	18	19	17	17	18
LMW*n*-acids/HMW*n*-acids^c^	3.12	2.36	63.67	6.40	3.07
CPI *n*-carboxylic acids (c_8_–C_28_)^d^	7.40	4.34	28.85	3.68	5.74
*n*-alkanols	0.35	0.83	0.56	0.68	0.42
ACL *n*-alkanols^b^	21	20	20	20	20
LMW*n*-alkanols/HMW*n*-alkanols^c^	0.70	0.98	0.92	0.98	0.98
CPI *n*-alkanols (c_11_–C_28_)^d^	6.71	4.38	3.45	4.29	8.25
cholesterol^k^	0.01	0.02	0.01	0.03	0.01
stigmastanol^l^	0.22	0.35	0.13	0.15	0.11
β-sitosterol^m^	0.02	0.03	0.02	0.01	0.01
Σ c_32_+C_34_ wax ester	n.d.	0.04	0.01	0.03	n.d.

^a^ Sum of mono-, di-, trimethyl *n*-alkanes.

^b^ Average chain length of C_14_–C_27_ *n*-alkanes; C_8_–C_28_ *n*-carboxylic acids and C_11_–C_28_ *n*-alkanols. ACL_*i-n*_ = Σ(*i*·*X_i_* + … + *n*·*X_n_*)/Σ*X_i_* + … + *X_n_*), where *X* is concentration (van Dongen *et al.,* 2008).

^c^ Low molecular weight (LMW) over high molecular weight (HMW). LMW is the sum of C_14_–C_20_ (*n*-alkanes), C_8_–C_20_ (*n*-carboxylic acids), and C_11_–C_20_ (*n*-alkanols). HMW is the sum of C_21_–C_27_ (*n*-alkanes), C_21_–C_28_ (*n*-carboxylic acids), and C_21_–C_28_ (*n*-alkanols).

^d^ Carbon preference index, CPI_*i-n*_ = ½Σ(*X_i_* + *X_i_*_+2_ + … + *X_n_*)/Σ(*X_i_*_−1_ + *X_i_*_+1_ + … + *X_n_*_−1_) + ½Σ(*X_i_* + *X_i_*_+2_ + … + *X_n_*)/Σ(*X_i_*_+1_ + *X_i_*_+3_ + … + *X_n_*_+1_), where *X* is concentration.

^e^ 2,6,10,14-tetramethyl-pentadecane.

^f^ 2,6,10,14-tetramethyl-hexadecane.

^g^ Sum of monounsaturated *n*-carboxylic acids (C_16_ and C_18_).

^h^ Sum of *iso-* and *anteiso- n*-carboxylic acids (C_14_–C_18_).

^i^ Sum of monomethyl C_22_ and C_24_ *n*-carboxylic acids.

^j^ Sum of all carboxylic acids (saturated, unsaturated, dicarboxylic, *iso/anteiso-* and monomethyl-).

^k^ Cholest-5-en-3b-ol.

^l^ 24-ethyl-5a-cholest-22-en-3b-ol.

^m^ 4-Ethylcholest-5-en-3β-ol.

n.d. = not detected.

**Table 2.** Concentration (μg·g^−1^) of *Normal* (*i.e.,* Straight-Chain) and Branched Alkanes in the Dallol Samples

*Alkanes*	*Abbreviation*^a^	*D6*	*D7*	*D8*	*D10*	*D11*
Tetradecane	C_14_	0.004	0.022	0.004	0.01	0.006
Dodecane. 5.8-dimethyl-	DiM-C_12_	0.002	0.005	0.005	0.005	0.004
Dodecane. 2.6.11-trimethyl	TriM-C_12_	0.015	0.071	0.022	0.055	0.019
Tridecane. 3-methyl-	MM-C_13_	0.002	0.016	0.012	0.016	0.004
Pentadecane	C_15_	0.006	0.027	0.009	0.017	0.009
Tetradecane. 4-methyl-	MM-C_14_	0.018	0.090	0.025	0.055	0.041
Hexadecane	C_16_	0.009	0.039	0.011	0.021	0.011
Pentadecane. 2.6.10.14-tetramethyl^b^	TetraM-C_15_	0.052	0.102	0.062	0.073	n.d.
Hexadecane. 6-methyl	MM-C_16_	0.002	0.040	n.d.	n.d.	0.033
Heptadecane	C_17_	0.012	0.045	0.014	0.028	0.015
Hexadecane. 2.6.10.14-tetramethyl^c^	TetraM-C_16_	n.d.	0.071	0.032	0.021	n.d.
Pentadecane. 3.6.11-trimethyl-	TriM-C_15_	0.004	0.004	0.003	0.006	0.005
Octadecane	C_18_	0.015	0.059	0.019	0.042	0.027
Nonadecane	C_19_	0.008	0.041	0.016	0.024	0.019
Eicosane	C_20_	0.009	0.028	0.013	0.022	0.017
Eicosane. 2-methyl	MM-C_20_	0.010	0.029	0.026	0.024	0.016
Heneicosane	C_21_	0.006	0.027	0.008	0.020	0.013
Docosane	C_22_	0.006	0.026	0.008	0.018	0.011
Tricosane	C_23_	0.005	0.024	0.006	0.015	0.010
Tetracosane	C_24_	0.003	0.023	0.007	0.012	0.009
Pentacosane	C_25_	n.d.	0.022	0.006	0.009	0.009
Hexacosane	C_26_	n.d.	0.021	n.d.	0.008	0.008
Heptacosane	C_27_	n.d.	0.018	n.d.	0.008	0.008
Σ br-alkanes^d^		0.105	0.428	0.187	0.255	0.122
Σ *n*-alkanes		0.083	0.422	0.121	0.254	0.172

^a^ C_*x*_ is used to denominate the linear and saturated (*normal*) alkanes; MM, DiM, TriM, and TetraM stand for mono-, di-, tri- and tetramethyl alkanes.

^b^ Pristane.

^c^ Phytane.

^d^ Sum of branched alkanes.

n.d. = not detected.

The *n*-carboxylic acids were measured at concentrations between 0.68 and 1.77 μg·g^−1^ (Tables 1 and 3), with chain lengths ranging from C_8_ to C_28_ (ACL ≤19). The straight-chained were the most abundant carboxylic acids, whereas unsaturated, branched, or dicarboxylic acids were scarcer (Table 1 and Fig. 4b). The molecular distribution of the majority *n*-carboxylic acids showed a clear dominance of the C_16_ and C_18_ peaks, with secondary peaks at C_12_, C_14_, and C_22_ (Fig. 4b). The resulting ratio of LMW over HMW carboxylic acids ranged from 2.3 to 63 (Table 1). Carboxylic moieties with different unsaturations (mono- and di-) were observed at C_16_, C_17_, and C_18_ at concentrations from 0.10 to 0.15 μg·g^−1^ (Tables 1 and 3). Acids with two carboxyl groups (*i.e.,* dioic or dicarboxylic acids) were also found from C_8_ to C_10_ at small concentrations ranging from 0.01 to 0.02 g·g^−1^ (Table 1). Branched *n*-carboxylic acids with *iso* and *anteiso* configuration (*i/a*) were detected between C_14_ and C_18_ at concentrations from 0.01 to 0.05 μg·g^−1^ (Table 1), with predominance of the *i*/*a*-C_15_ and *i*/*a*-C_17_ (Fig. 4b). Other branched *n*-carboxylic acids included monomethyl C_22_ and C_24_ acids (Table 1).

**Table 3.** Concentration (μg·g^−1^) of Carboxylic Acids (Saturated, Unsaturated, Branched, and Dicarboxylic) in the Dallol Samples

*Carboxylic acids*	*Abbreviation*^a^	*D6*	*D7*	*D8*	*D10*	*D11*
Octanoic acid	C_8_	0.011	0.014	0.001	0.001	0.011
Nonanoic acid	C_9_	0.003	0.028	0.002	0.002	0.003
Decanoic acid	C_10_	0.013	0.018	0.001	0.001	0.013
Undecanoic acid	C_11_	0.002	0.001	0.001	n.d.	0.002
Octanedioic acid	di-C_8_	0.004	0.003	0.004	0.005	0.004
Dodecanoic acid	C_12_	0.024	0.070	0.010	0.008	0.02
Nonanedioic acid	di-C_9_	0.014	0.011	0.005	0.003	0.005
Tridecanoic acid	C_13_	0.002	0.002	0.010	0.019	0.002
Decanedioic acid	di-C_10_	0.002	0.001	0.002	0.003	0.002
Tridecanoic acid, 12-methyl-	isoC_14_	n.d.	0.002	0.002	n.d.	n.d.
cis-9-Tetradecenoic acid	anteisoC_14_	n.d.	0.004	n.d.	n.d.	n.d.
Tetradecanoic acid	C_14_	0.019	0.050	0.012	0.071	0.019
Methyl 13-methyltetradecanoate	isoC_15_	0.003	0.003	n.d.	n.d.	0.003
Tetradecanoic acid, 12-methyl	anteisoC_15_	n.d.	0.005	n.d.	0.001	0.001
Pentadecanoic acid	C_15_	0.01	0.058	0.013	0.041	0.010
Pentadecanoic acid, 13-methyl	isoC_16_	0.001	0.002	n.d.	0.001	n.d.
Pentadecanoic acid, 14-methyl	anteisoC_16_	n.d.	0.002	n.d.	0.002	0.002
9-Hexadecenoic acid (Z)	C_16:1 (ω7)_	0.017	0.022	n.d.	0.001	0.020
Hexadecanoic acid	C_16_	0.27	0.413	0.374	0.280	0.174
Hexadecanoic acid, 15-methyl	isoC_17_	0.006	0.011	0.004	n.d.	0.004
Methyl 8-heptadecenoate	anteisoC_17_	0.003	0.009	n.d.	n.d.	0.003
Heptadecanoic acid	C_17_	0.017	0.021	n.d.	0.046	0.017
Heptadecanoic acid, 16-methyl	isoC_18_	n.d.	0.012	0.011	0.018	n.d.
9,12-Octadecadienoic acid (Z,Z)	C_18:2 (ω6,9)_	0.021	0.023	0.015	0.011	0.021
9-Octadecenoic acid (Z)-	C_18:1 (ω9)_	0.056	0.062	0.065	0.093	0.056
8-Octadecenoic acid (E)-	C_18:1 (ω10)_	0.024	0.042	0.035	0.024	0.004
Octadecanoic acid	C_18_	0.244	0.463	0.322	0.237	0.214
Nonadecanoic acid	C_19_	0.006	0.030	n.d.	0.037	0.006
Octadecanoic acid, 10-oxo-	Oxo-C_19_	0.002	0.002	n.d.	n.d.	0.002
Eicosanoic acid	C_20_	0.025	0.076	0.018	0.019	0.025
Heneicosanoic acid	C_21_	0.023	0.057	n.d.	0.015	0.023
Methyl 11-docosenoate	MM-C_22_	n.d.	0.004	0.004	0.010	n.d.
Docosanoic acid	C_22_	0.052	0.017	0.012	0.037	0.042
Tricosanoic acid	C_23_	0.020	0.074	n.d.	0.011	0.02
Tetracosanoic acid	C_24_	0.053	0.146	n.d.	0.030	0.034
Methyl,22-methyl-tetracosanoate	MM-C_24_	n.d.	0.016	0.007	0.015	0.010
Pentacosanoic acid	C_25_	0.013	0.029	n.d.	0.004	0.013
Hexacosanoic acid	C_26_	0.036	0.115	n.d.	0.021	0.026
Heptacosanoic acid	C_27_	0.003	0.020	n.d.	n.d.	0.003
Octacosanoic acid	C_28_	0.007	0.070	n.d.	0.001	0.007
Σ *n*-carboxylic acids		0.853	1.772	0.776	0.881	0.684
Σ unsaturated carboxylic acids		0.118	0.149	0.115	0.129	0.101
Σ dicarboxylic acids		0.020	0.015	0.011	0.011	0.011
Σ *iso/anteiso* carboxylic acids		0.013	0.050	0.017	0.022	0.013
Σ MM carboxylic acids		n.d.	0.020	0.011	0.025	0.010

^a^ C_*x*_ is used to denominate the linear and saturated (*normal*) carboxylic acids, where *x* is the number of carbons; C_*x:y*_ is used to denominate the unsaturated carboxylic acids, where *x* is the number of carbons and *y* the number of unsaturations; MM stands for mono-methyl chains and Oxo for the ketoacids.

**Table 4.** Concentration (μg·g^−1^) of *Normal* Alkanols in the Dallol Samples

*Alkanols*	*Acronym*	*D6*	*D7*	*D8*	*D10*	*D11*
Undecanol	C_11_	0.002	0.005	0.005	0.005	0.002
Dodecanol	C_12_	0.010	0.065	0.033	0.061	0.018
Tridecanol	C_13_	0.002	0.008	0.016	0.016	0.002
Tetradecanol	C_14_	0.018	0.060	0.040	0.070	0.038
Pentadecanol	C_15_	0.002	0.004	0.004	0.004	0.004
Hexadecanol	C_16_	0.028	0.070	0.035	0.030	0.027
Heptadecanol	C_17_	0.010	0.031	0.013	0.021	0.010
Octadecanol	C_18_	0.049	0.119	0.085	0.119	0.072
Nonadecanol	C_19_	0.002	0.002	0.005	0.002	0.002
Eicosanol	C_20_	0.022	0.070	0.050	0.030	0.039
Heneicosanol	C_21_	0.003	0.038	0.018	0.022	0.006
Docosanol	C_22_	0.071	0.140	0.101	0.111	0.082
Tricasanol	C_23_	0.009	0.012	0.012	0.012	0.009
Tetracosanol	C_24_	0.052	0.070	0.050	0.070	0.038
Pentacosanol	C_25_	0.006	0.033	0.023	0.023	0.006
Hexacosanol	C_26_	0.025	0.051	0.029	0.031	0.025
Heptacosanol	C_27_	0.012	0.016	0.006	0.016	0.012
Octacosanol	C_28_	0.025	0.039	0.031	0.039	0.025
Σ *n*-Alkanols		0.348	0.833	0.556	0.682	0.417

Straight-chained alkanols (*i.e., n*-alkanols) were the second most abundant lipids (0.35–0.83 μg·g^−1^) in the Dallol samples (Fig. 3 and Table 4). They showed even distributions (carbon preference index, CPI = 3.4–8.2) from C_11_ to C_28_ (ACL ≤21), with dominance of the C_22_ and C_18_ congeners and minority but important presence of the C_12_-C_14_ and C_24_-C_26_-C_28_ peaks (Fig. 4c). In the same ion (*i.e., m/z* = 75), three sterols belonging to the cholesterol and phytosterol (stigmastanol and β-sitosterol) classes were also detected (Fig. 4c), with stigmastanol being the most abundant sterol (0.11–0.35 μg g^−1^; Table 1).

Finally, a few wax esters (*i.e.,* hexadecyl hexadecanoate, C_32_; and octadecyl hexadecanoate, C_34_) were measured in the polar fraction (ions *m/z* 257, 480; and *m/z* 257, 508, respectively) at low concentrations (≤0.04 μg g^−1^) in the D7, D8, and D10 samples (Table 1).

## 4. Discussion

### 4.1. Bulk geochemistry in the Dallol hot springs

The mineralogical composition of the Dallol samples explained the color range observed in the sampled precipitates. The light yellow and whitish tones (Fig. 1d, 1e) reflected the majority content of halite and the presence of freshly exposed bromian chlorargyrite, whereas the brown and orangish tones revealed the presence of wurtzite and oxidized chlorargyrite. The high halite content is typical of evaporitic deposits and common in hypersaline environments such as the Atacama Desert (*e.g.,* Fernández-Remolar *et al.,* 2013; Sánchez-García *et al.,* 2018). The abundance of halite is known to play a role in the preservation of organics including microbial biosignatures, a process that has been described in different hypersaline (Fernández-Remolar *et al.,* 2013; Schinteie and Brocks, 2016; Cheng *et al.,* 2017) and hydrothermal (Pancost *et al.,* 2005; Zhang *et al.,* 2007) systems. In hypersaline and hyperarid environments similar to Dallol, a wide variety of preservation strategies have been reported such as entrapment in salt crystals (*e.g.,* de los Ríos *et al.,* 2010; Cheng *et al.,* 2017), xeropreservation (Wilhelm *et al.,* 2017; Sánchez-García *et al.,* 2018), or cellular modifications as survival mechanisms upon stress (Morgan *et al.,* 2006). Hydrothermal minerals such as bromian chlorargyrite and wurtzite are known to form in acidic and saline hydrothermal solutions that undergo surface deposition (Nickel, 2002).

The low values of TOC in the five Dallol samples (Fig. 2a) illustrated the limited content of organic matter in a system where the polyextreme conditions hamper the development of life. Dallol is an unvegetated region, where the intense solar radiation, elevated temperatures, hyperacidity, and high salinity make the hydrothermal substrate fairly inhospitable. In fact, no lichens, higher plants, or native animals were observed in the place during the sampling period. The only form of life capable of adapting and thriving in such a hostile environment is expected to be majorly prokaryotic (*i.e.,* bacteria, archaea), with potential presence of acidophilic fungi, and to occupy specific niches. The diversity of geochemical conditions existing in pools and fumaroles, where hydrothermal fluids and surrounding evaporites are characterized by abrupt gradients of temperature, pH, redox potential, oxygen concentration, mineral content, and metal availability, provides a variety of niches for the microbial communities, in a similar way as in deep-sea hydrothermal vents (McCollom and Shock, 1997).

The δ^13^C ratios (−22.6‰ to −25.9‰) measured in the Dallol samples (Fig. 2b) are in the range of those reported in similar geothermal environments such as the Obsidian Pool in the Yellowstone National Park (Schuler *et al.,* 2017), El Tatio geyser field in Chile (Sánchez-García *et al.,* 2019), Uzon Caldera in Kamchatka (Russia; Burgess *et al.,* 2011), or California (Eagleville) and Nevada (Paradise Valley and Crescent Valley) hot springs (Zhang *et al.,* 2007), and suggest a dominant autotrophic fingerprint. According to kinetic and thermodynamic methods, microorganisms using the acetyl coenzyme A (CoA) pathway express the largest fractionation of ^13^C during fixation of CO_2_ (Δδ^13^C from 15‰ to 36‰), whereas those using the Calvin cycle display somewhat less discrimination (Δδ^13^C from 11‰ to 26‰) (Hayes, 2001). Fractionations even lower occur with CO_2_ incorporations using the reductive citric acid (Δδ^13^C from 3‰ to 13‰) or hydroxypropionate (Δδ^13^C from 2‰ to 13‰) cycles (van der Meer *et al.,* 2000; Hayes, 2001). In the Dallol Hot Springs, three potential carbon substrates were considered for the autotrophs to grow. Together with atmospheric CO_2_ and dissolved inorganic carbon (DIC), dissolved CO_2_ is an important source of carbon in the hyperacidic springs generated from mixing of CO_2_-rich ascending magmatic fluids, boiling meteoric water, and seawater trapped in the evaporitic sequence (Kotopoulou *et al.,* 2019). Assuming a mean δ^13^C value for atmospheric CO_2_ of −8‰ (Graven *et al.,* 2017), a δ^13^C value ranging from −2.8‰ to −6.2‰ for dissolved CO_2_ as measured by others on CO_2_-emitting fumaroles from the Dallol region (Darrah *et al.,* 2013), and a DIC δ^13^C value of −2.5‰ as that measured on DIC from similar hot springs (Obsidian Pool) in the Yellowstone National Park (Schuler *et al.,* 2017), we considered that fractionation upon biological incorporation of carbon in the Dallol hydrothermal system ranged from 15‰ to 23‰ relative to the three potential carbon substrates. These fractionation values are in the range of those involved in the Calvin cycle (*e.g.,* Cyanobacteria or α-, β-, and γ-Proteobacteria; Hayes *et al.,* 1983; Bar-Even *et al.,* 2011) and in the lower edge of those using the CoA pathway for CO_2_ incorporation (*e.g.,* some Firmicutes, methanogenic Euryarchaeota, or chemolithotrophic Planctomycetes; Bar-Even *et al.,* 2011; Havig *et al.,* 2011). Interestingly, in microbial mats constructed by *Chloroflexus* in conjunction with Cyanobacteria, where the former grows photoheterotrophically by consuming cyanobacterial photosynthate, organic matter may show isotopic signatures more typical of the Calvin cycle (*e.g.,* −23.5‰) than of the hydroxypropionate cycle characteristically used by Chloroflexi (van der Meer *et al.,* 2001). Members of Chloroflexi, Proteobacteria, Firmicutes, Euryarchaeota, Bacteroidetes, Actinobacteria, or Acidobacteria have been previously described in commercial salt from the Dallol hydrothermal system (Gibtan *et al.,* 2016, 2017).

As for the sulfur isotopic composition, the enriched δ^34^S signatures measured both on sulfate (6.5‰ to 11.7‰) and total sulfur (8.2‰ to 20.5‰) suggested the participation of sulfate-reducing bacteria in the microbial community of Dallol. Bacterial sulfate reduction produces isotopic fractionation during the microbial sulfur cycle, which results in enriched δ^34^S values in sulfate and total sulfur in contrast to the lighter δ^34^S values of sulfide (Canfield, 2001). In Dallol, the presence of sulfate as one of the few inorganic ions detected in the evaporitic samples (14 ± 0.83 mg/g) would ensure the substrate required by sulfate-reducing bacteria to grow.

### 4.2. Lipid biosignatures in the Dallol evaporites

The lipid analysis of the Dallol samples revealed a dominant microbial signature. The three major lipid families (*i.e., n*-alkanes, *n*-carboxylic acids, and *n*-alkanols) showed a characteristic even-over-odd distribution (Fig. 4). In the nonpolar fraction (Fig. 4a), the relative abundance of LMW *n*-alkanes (≤C_20_) with maximum at C_18_ (ACL ≤20) and the prevailing even character (CPI ≤1) (Table 1) reflected microbial signatures (Grimalt and Albaigés, 1987; Meyers and Ishiwatari, 1993; Volkman *et al.,* 1998). This was supported by the relative abundance of monomethyl alkanes among the detected branched alkanes (Table 2), components that have been described in bacterial communities (including Cyanobacteria) from hydrothermal (Cady and Farmer, 1996; Campbell *et al.,* 2015b) and hypersaline (Dembitsky *et al.,* 2001) environments. Other microbial biomarkers found in the Dallol samples were the isoprenoids pristane and phytane (Fig. 4a). These compounds are mainly originated from phytol, the esterifying alcohol of phototrophic chlorophylls (Didyk *et al.,* 1978), which degradation gives rise to pristane or phytane in the presence or absence of oxygen, respectively (Peters *et al.,* 2005). In addition, phytane may have alternative sources such as archaeols (Brocks and Summons, 2003) or tocopherols (E vitamins; Goossens *et al.,* 1984). In Dallol, the lack of autochthonous vegetation led us to consider different phototrophic sources of pristane and phytane such as cyanobacteria or bacteria containing bacteriochlorophylls *a* and *b* (Peters and Moldowan, 1993). Although the growth of cyanobacteria and other phototrophic bacteria is seriously hampered at temperatures higher than 73°C (Ward *et al.,* 1989; Miller and Castenholz, 2000) and pH values below ∼4 in the case of cyanobacteria (Cirés *et al.,* 2017), in hydrothermal systems such as Dallol these parameters typically change with abrupt gradients in short distance (McCollom and Shock, 1997) and temporal ranges, with active spring sites going inactive and new springs emerging in new locations in the range of days (Kotopoulou *et al.,* 2019). This dynamicity and variability in the Dallol hydrothermal system make it possible to find niches of lower temperature and higher pH, where phototrophic microorganisms are able to thrive in a polyextreme environment otherwise unsuitable for photosynthetic microorganisms. To a lesser extent, pristane and phytane could also come from the degradation of phytol present in vegetal masses extending in neighboring areas, where wind may have played a role in transporting the allochthonous material. Woods and biomass extensions spreading about 7 km away from the hydrothermal zone could provide the vegetal material to be aerially introduced into the hydrothermal system. In either case, the relative abundance of pristane relative to phytane would reflect the predominance of oxic conditions in the degradation of phytol, which is consistent with the prevailing oxicity existing in evaporitic environments (Chong-Díaz *et al.,* 1999) such as Dallol. On the other hand, the lower concentration of phytane relative to pristane could be due to a different origin than phytol for this compound (*i.e.,* archaeols or tocopherols).

**FIG. 5.**
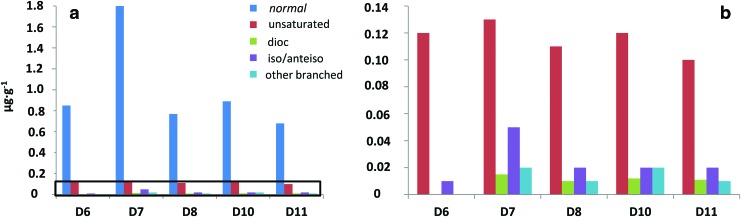
Qualitative composition of the acidic fraction isolated from the Dallol evaporite samples (**a**), with a zoom view on the area within the black inlet (**b**). In the legend, *“normal”* stands for straight-chain acids, “unsaturated” for mono- and diunsaturated acids, “dioic” for dicarboxylic acids, “iso/anteiso” for branched acids with methyl groups in *iso* and *anteiso* positions, and “other branched” for other methylated non *iso*/*anteiso* carboxylic acids.

The microbial signature was also reflected in the distribution of carboxylic acids. First, the majoritarian straight-chain carboxylic acids showed a distinct even character (CPI = 3.7–28) and a predominance of short chains (ACL <19) with maximum peaks at C_16_ and C_18_ (Fig. 4b). These even and short distributions are typically associated with microbial sources (Cranwell, 1974), as the majority of bacteria have a simple carboxylic acid composition with significant proportion of myristic (C_14_), palmitic (C_16_), and stearic (C_18_) acids (Kaneda, 1991). Second, unsaturations were ubiquitously detected on the C_16_ (C_16:1_ ω7) and C_18_ (C_18:1_ ω9, C_18:1_ ω10, and C_18:2_ ω6,9) carboxylic acids (Fig. 4b and Table 3). The detection of these and other polyunsaturated acids in Octopus Spring (temperature of 87°C and pH of 8.3) in Yellowstone (USA) was related to the presence of thermophilic bacteria from the orders Aquificales and Thermotogales (Jahnke *et al.,* 2001). In particular, the C_18:1_ (ω9) acid was recognized in thermophilic microorganisms within the Chloroflexi phylum (*i.e., Thermomicrobium*) (Jahnke *et al.,* 2001; Kaur *et al.,* 2015). Third, the Dallol acidic fraction contained low concentrations of dicarboxylic acids ranging from C_8_ to C_10_ (Fig. 5b). Comparable distributions of short dicarboxylic acids (C_6_–C_10_) in the Octopus Spring were described as core lipids of Thermotogales members (Carballeira *et al.,* 1997; Jahnke *et al.,* 2001). Finally, the ubiquitous detection of short chains (C_14_–C_18_) of *iso*/*anteiso* carboxylic acids in the Dallol samples (Fig. 4b) reinforced the microbial hypothesis. The branched carboxylic acids, in particular the *iso*/*anteiso* C_15_ and C_17_ pairs, are typically associated with bacterial sources (Kaneda, 1991), being particularly abundant in sulfate-reducing bacteria (Langworthy *et al.,* 1983). They were found in thermophilic bacteria from the *Thermus* and *Meiothermus* genera in cultured strains (Nobre *et al.,* 1996; Yang *et al.,* 2006) and in thermophilic bacteria from the Octopus Springs in Yellowstone (Jahnke *et al.,* 2001). The *iso*/*anteiso* C_15_ and C_17_ pairs have also been described in other hot springs, for example, in New Zealand (Kaur *et al.,* 2015), as well as in microbial mats from the Shark Bay in Australia (Pagés *et al.,* 2015), or in ooids (small and spheroidal, concentric layered sedimentary grains of calcium carbonate) from Bahamas and Australia (Summons *et al.,* 2013). Other branched carboxylic acids such as the *iso*-C_18_ were described in certain Chloroflexi members in hydrothermal systems in New Zealand and in Octopus Spring (Jahnke *et al.,* 2001; Kaur *et al.,* 2015), as well as in the *Desulfobacter* spp. (Dowling *et al.,* 1988; Summons *et al.,* 2013). The detection of wax esters of 32 and 34 carbons in three of the five Dallol samples (*i.e.,* D7, D8, and D10), although at small concentrations (Table 1), also supported the presence of anoxygenic phototroph *Chloroflexus,* as it was described in hydrothermal systems of New Zealand (Campbell *et al.,* 2015b). Thermophiles such as *Chloroflexus* and *Roseiflexus* typically dwell in microbial mats together with Cyanobacteria, growing photo-heterotrophically by consuming the cyanobacterial photosynthate (van der Meer *et al.,* 2000). In Dallol, the combined detection of monomethyl alkanes, C_18:1_ and C_18:2_ carboxylic acids, pristine and phytane, and wax esters suggests the presence of Cyanobacteria in the Dallol evaporites, likely forming Cyanobacteria-*Chloroflexus* mats based on the reported presence of *Chloroflexus* in commercial salts from the area (Gibtan *et al.,* 2017).

In addition to the microbial character dominating the Dallol signatures, some contribution of vegetal sources was inferred from the presence of secondary groups of HMW *n-*carboxylic acids (Fig. 4b) and *n-*alkanols (Fig. 4c) of even character and maximum peaks at C_22_–C_28_. These types of distributions are typically observed in organic matter from macrophytes or higher plants (Eglinton and Hamilton, 1967; Feng and Simpson, 2007). Their identification in Dallol, together with certain sterols (*i.e.,* stigmastanol or β-sitosterol), was consistent with the presence of vegetal vestiges. Sterols are ubiquitous constituents in all eukaryotic organisms, where stigmastanol or β-sitosterol are almost exclusively produced by higher plants (Patterson and Nes, 1991; Goad and Akihisa, 1997), whereas cholesterol is majorly produced from animals (Volkman, 1986). The vegetal and animal signatures in Dallol were explained by external inputs from nearby areas of woods or human settlements. In the nearby Gaet'ale spring, 3.8 km southeast of Dallol, Master (2016) described the presence of vegetal and human debris, washed in by rains from the woods and sparse villages located in the highlands. These runoff episodes are common in this area given the low altitude of the floor in the Danakil Depression. In addition, the presence of animals (at least birds and insects) in the region (Master, 2016) may also explain the observation of animal biosignatures. Altogether, the molecular distribution of alkanes, carboxylic acids, alkanols, sterols, and wax esters revealed the presence of biological vestiges in the Dallol evaporite samples. In particular, microbial sources appeared to be dominant according to the relative abundance of the short over the long *n-*alkanes, *n-*carboxylic acids, or *n-*alkanols (ACL ≤21; Table 1). Despite the inhospitability of the Dallol Hot Springs, at least some extremophilic microorganisms appear to be resistant enough to endure the extremely low pH and high temperatures and live (past or present) in the polyextreme environment.

### 4.3. Molecular indications of active or recent metabolisms

The molecular distribution of the diverse lipid families manifested a generalized presence of microbial vestiges in the Dallol evaporites. While a definitive distinction between presently active metabolisms or fossilized biological fingerprints cannot be accomplished, we argue here a couple of observations that led us to consider that active or recent metabolisms dominate in Dallol. The relative abundance of functionalized (*i.e., n*-carboxylic acids and *n*-alkanols) versus saturated (*i.e., n*-alkanes) hydrocarbons (Fig. 3) was indicative of extant communities or recent biogenesis (Simoneit *et al.,* 1998). *n*-Carboxylic acids and *n*-alkanols are labile lipids that tend to be rapidly destroyed during diagenesis (Brocks and Summons, 2004) by cleavage of alkyl chains that produces *n-*alkanes without odd-over-even predominance (Killops and Killops, 2005). Without active metabolisms, the presence of functionalized groups tends to be minority relative to the saturated moieties. In the Dallol samples, *n*-carboxylic acids and *n*-alkanols were not only considerably more abundant than *n*-alkanes (*i.e.,* two to four times), but the latest showed beside an atypical even distribution pattern with maximum at C_18_ that coincided with the microbial-diagnostic C_18_ maximum peaks in the carboxylic (Fig. 5b) and hydroxyl (Fig. 5c) fractions. Given the short residence time of the labile *n*-carboxylic acids and *n*-alkanols in most environments, their detection in Dallol at such proportions suggested that they derive from either extant biomass or exceptionally well-preserved fossil lipids.

On the other hand, the relative enrichment of even compounds in the three majority families, even the *n*-alkanes, supports the hypothesis of active metabolisms or very good preservation. *n*-Alkanes derived from biological sources typically show preference for odd (plants; Eglinton and Hamilton, 1967) or even (microorganisms; Meyers and Ishiwatari, 1993) carbons, whereas the absence of even/odd patterns may reveal an advanced diagenesis (Killops and Killops, 2005) or abiotic origin (McKay, 2004) of those lipids. In Dallol, CPI values lower than or equal to the unit in the *n*-alkanes (Table 1) denote organic matter decay by either active or past microbial metabolisms.

In sum, the lipid analysis enabled us to identify molecular evidence of life (mostly microbial) in the polyextreme environment of Dallol Hot Springs. The abundance of functionalized relative to saturated hydrocarbons pointed to present or recently active metabolisms producing typical microbial signatures. The preservation of functionalized-fossil lipids by encasing inside salt precipitation is another plausible way (Conner and Benison, 2013) to explain the “fresh” biosignatures detected in the halite-rich hydrothermal system. This is the first study reporting the detection of (present or recent) life in the Dallol evaporitic system. These findings are relevant for constraining the limits of life and have implications for the search for extraterrestrial life. The existence of viable life in the polyextreme environment of Dallol expands our understanding of the limits of life and supports the habitability on analogous environments in other planetary bodies (*e.g.,* martian geological sites; Klingelhofer *et al.,* 2004; Bishop *et al.,* 2015). Whereas further investigation is needed to more comprehensively identify the microbial communities associated to the hydrothermal and evaporitic substrates, the present study constitutes the first biogeochemical approach to describe the Dallol polyextreme environment. In the future, additional analyses such as that of phospholipid fatty acids will be conducted to assess the presence of presently operative metabolisms.

## 5. Conclusion

This study is the first to show lipid molecular evidence of life in hydrothermal deposits of the Dallol Hot Springs, a geothermal extreme environment in eastern Ethiopia combining hypersalinity, acidity, and high water temperatures. A lipid biomarker approach was used to elucidate preserved biosignatures in a polyextreme environment with interest for understanding the limits of life. The abundance of low- over high-molecular-weight chains (*n*-alkanes, *n*-carboxylic acids, and *n*-alkanols) together with the detection of a number of bacterial-diagnostic compounds documented the predominance of microbial lipids in the Dallol samples. We hypothesize that the microbial signatures most likely correspond to present or recently active metabolisms, according to the large proportion of functionalized hydrocarbons and the distinct even-over-odd pattern of all lipid families, including the *n*-alkanes. The observed molecular distributions suggest that, despite the inhospitability of the Dallol Hot Springs, there are at least some microorganisms capable of living in such extreme pH, temperature, and salinity conditions. These findings contribute to elucidate where the limits of life may be and where it may be interesting to search for life in analogous extraterrestrial environments. Whereas further investigation is needed to identify comprehensively the microbial communities associated to the hydrothermal and evaporitic substrates, the present study constitutes the first biogeochemical approach to identify (present or recent) life vestiges in the Dallol polyextreme environment. Future work is planned to combine proteomics and compound specific-isotopic analysis for achieving a metabolic and phylogenetic characterization of the microbial community inhabiting Dallol.

## Supplementary Material

Supplemental data

## Notes

Associate Editor: Jack Mustard

Supplementary Information: The graphical abstract and Supplementary Fig. S1 may be found in the online version of this article at www.liebertonline.com/ast

## Abbreviations Used

ACL: average chain length
CPI: carbon preference index
DIC: dissolved inorganic carbon
FAMEs: fatty acid methyl esters
GC-MS: gas chromatography–mass spectrometry
HMW: high-molecular-weight
IC: ion chromatography
IRMS: isotope ratio mass spectrometry
LMW: low-molecular-weight
*m/z*: mass-to-charge ratios
PDB: Pee Dee Belemnite limestone standard
TLE: total lipid extract
TOC: total organic carbon
VCDT: Vienna-Canyon Diablo Troilite
XRD: X-ray diffractometer
