# Supplementary material for: Lipid Biomarker and Carbon Stable Isotope Survey on the Dallol Hydrothermal System in Ethiopia
Source: Astrobiology. 2019 Dec 4;19(12):1474–89. doi: 10.1089/ast.2018.1963 (PMC6921156; doi:10.1089/ast.2018.1963)

Figure S1. Analytical scheme of the total lipid extract fractionation and target lipid compounds in each fraction.

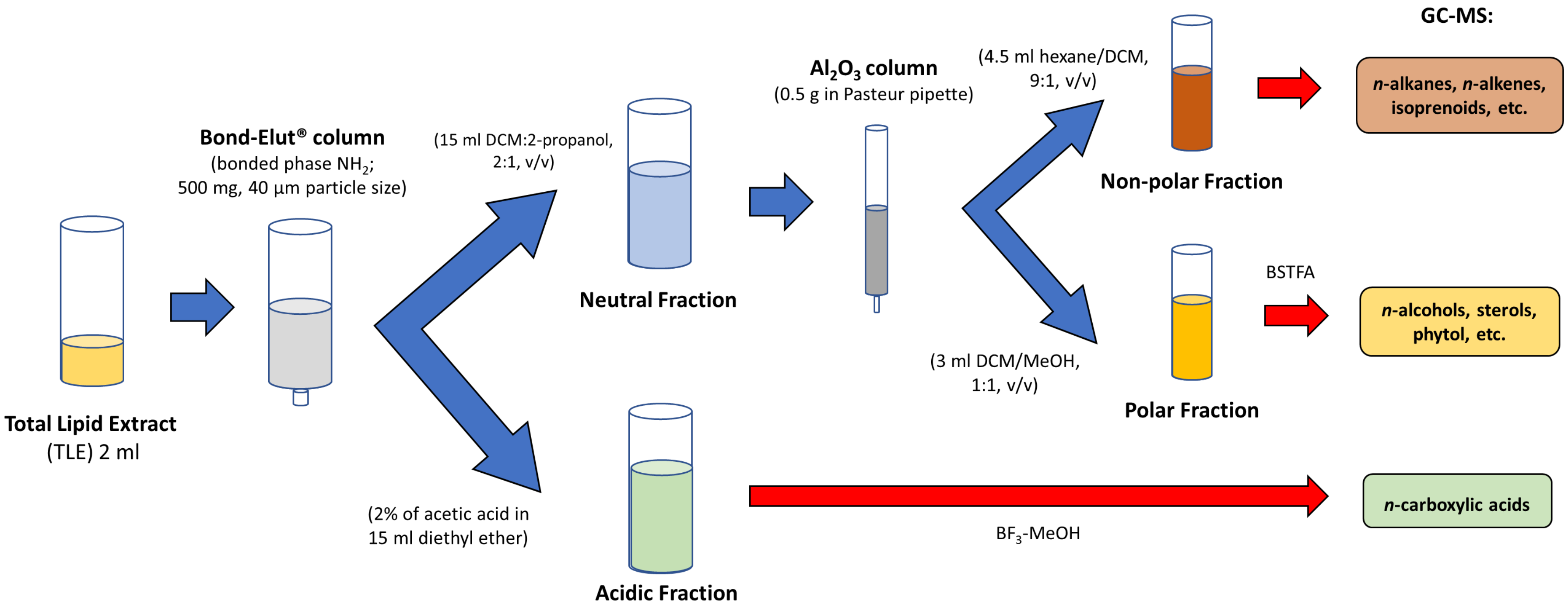

Supplement: Supplemental data [file Supp_Fig1.pdf]
